# Supplementary figures and images for: Breakthrough COVID-19 Infections after Booster SARS-CoV-2 Vaccination in a Greek Cohort of People Living with HIV during the Delta and Omicron Waves
Source: Biomedicines. 2024 Jul 19;12(7):1614. doi: 10.3390/biomedicines12071614 (PMC11274973; doi:10.3390/biomedicines12071614)

**Supplementary Figure S1.** Temporal trends in vaccination prescription according to dose

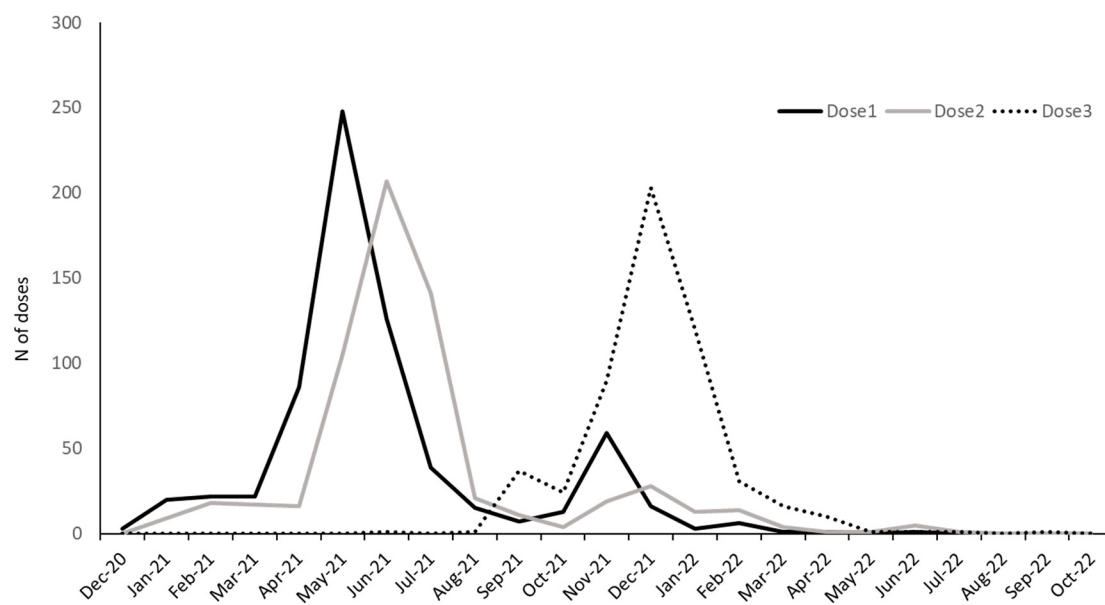

Supplement: Supplementary file 1 [file biomedicines-12-01614-s001.zip › biomedicines-3044021-supplementary.pdf]
